# Incomplete bowel obstruction caused by sigmoid colon cancer in an inguinal hernia: a case report

**DOI:** 10.1186/s40792-024-01874-1

**Published:** 2024-04-24

**Authors:** Hiroki Sujino, Hideki Gon, Yota Shimoda, Chie Takishita, Masanobu Enomoto, Shingo Tachibana, Kazuhiko Kasuya, Yuichi Nagakawa

**Affiliations:** 1 Department of Surgery, Toda Chuo General Hospital, 1-19-3 Honcho, Toda, Saitama 335-0023 Japan; 2 Department of Gastrointestinal and Pediatric Surgery, Tokyo Medical University, 6-7-1 Nishishinjuku, Shinjuku, Tokyo 160-0023 Japan

**Keywords:** Inguinal, Hernia, Incarceration, Sigmoid, Colon, Cancer, Bowel obstruction

## Abstract

**Background:**

Most colon cancers that develop in the intestinal tract within the inguinal hernia sac are identified by incarceration. However, treatment methods for these cases vary depending on the pathology. Cases showing perforation or abscess formation require emergency surgery for infection control, while cases with no infection generally involve oncological resection, with laparoscopic surgery also being an option. We encountered a case of Incomplete bowel obstruction secondary to sigmoid colon cancer within the hernial sac. We report the process leading to the selection of the treatment method and the surgical technique, along with a review of the literature.

**Case presentation:**

A 79-year-old man presented to our hospital complaining of a left inguinal bulge (hernia) and pain in the same area. The patient had the hernia for more than 20 years. Using computed tomography, we diagnosed an incomplete bowel obstruction caused by a tumor of the intestinal tract within the hernial sac. Since imaging examination showed no signs of strangulation or perforation, we decided to perform elective surgery after a definitive diagnosis. After colonoscopy, we diagnosed sigmoid colon cancer with extra-serosal invasion; however, we could not insert a colorectal tube. Although we proposed sigmoid resection and temporary ileostomy, we chose the open Hartmann procedure because the patient wanted a single surgery. For the hernia, we simultaneously used the Iliopubic Tract Repair method, which does not require a mesh. Eight months after the surgery, no recurrence of cancer or hernia was observed.

**Conclusions:**

We report a case of advanced sigmoid colon cancer with a long-standing inguinal hernia that later became incomplete bowel obstruction. Although previous studies have used various approaches among the available surgical methods for cancer within the hernial sac, such as inguinal incision, laparotomy, and laparoscopic surgery, most hernias are repaired during the initial surgery using a non-mesh method. For patients with inguinal hernias that have become difficult to treat, the complications of malignancy should be taken into consideration and the treatment option should be chosen according to the pathophysiology.

## Background

Colon cancer observed in the intestinal tract within the inguinal hernia sac is rare. Most patients with this disease visit the hospital because of incarceration of the intestinal tract within the hernial sac. Emergency surgery may be indicated in patients showing intestinal strangulation or perforation. In contrast, in patients without infection or intestinal ischemia, colonoscopy may be performed to determine the cause of incarceration, which varies depending on the situation. We encountered a case of incomplete bowel obstruction due to colon cancer in the hernial sac. We report the process leading to the selection of the treatment method and surgical technique, along with a review of the literature.

## Case presentation

A 79-year-old male patient presented to our hospital with a bulge and pain in the left inguinal region. The patient had been aware of the bulge in the left inguinal region for more than 20 years; however, it had been left untreated because it naturally reduced in the supine position. However, the left inguinal bulge had been incarcerated for several recent years. At the time of admission, the patient’s body temperature, blood pressure, and pulse rate were 36.6 °C, 112/56 mmHg, and 75 beats/min, respectively. Physical examination showed that the abdominal wall in the left inguinal region was swollen to the size of a child's head and was mildly tender upon palpation. No symptoms of peritoneal irritation were observed on the abdomen. Blood biochemical analyses showed chronic anemia (hemoglobin 9.8 g/dL, red blood cells 4.25 × 10^6^/µL, hematocrit 32.0%, serum iron 22 µg/dL), hypoalbuminemia (albumin: 2.6 g/dL), increased inflammatory reaction (white blood cell count 15,120/µL, C-reactive protein 3.15 mg/dL), and elevated tumor marker levels (carcinoembryonic antigen 6.7 ng/mL, cancer antigen 19–9: 20.3 U/mL). Plain computed tomography (CT) at admission revealed thickening of a part of the intestinal wall within the hernia sac, along with a large amount of feces in the intestinal tract proximal to the same area. No abscesses, free air, or signs of intestinal strangulation were observed. Based on these CT images, we diagnosed Incomplete bowel obstruction caused by a tumor in the intestinal tract pouch, and the patient was admitted on the same day. The absence of signs of strangulation or perforation in the CT findings, combined with the patient's lack of peritoneal irritation symptoms and presentation of watery stools, led us to plan elective surgery after a definitive diagnosis.

**Fig.1:**
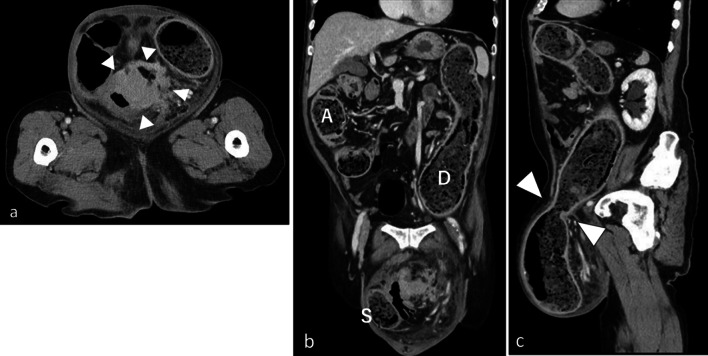
CT in the 2nd hospital days Wall thickening (arrow head) of the incarcerated intestine within the hernia was shown at axial view (**a**). Large amounts of faces in the ascending (A), descending (D) and sigmoid colon within the hernia (S) at coronal view (**b**). An enlarged hernia orifice (arrow head) was shown at sagittal view (**c**)

**Fig.2:**
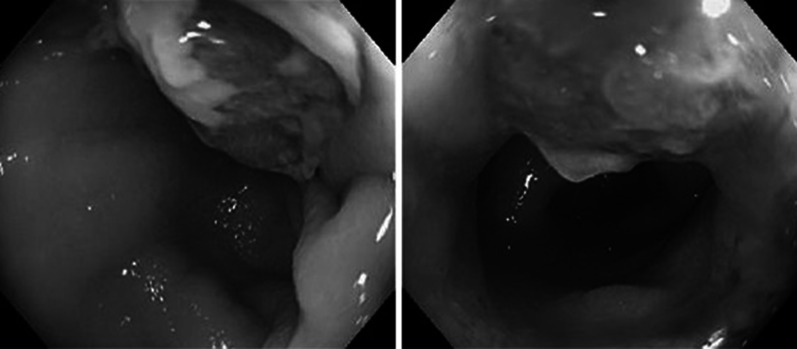
Colonoscopy in the 3rd hospital day. Advanced cancer was observed in sigmoid colon incarcerated within a hernia

Two days post-admission, the contrast-enhanced CT showed that the sigmoid colon within the hernia sac had wall thickening with contrast enhancement. No ascites was observed in the peritoneal cavity or hernia sac, leading us to suspect sigmoid colon cancer with extra serosal invasion but without peritoneal dissemination (Fig. 1). The next day, we performed colonoscopy to biopsy the lesion and insert a colorectal tube. The scope (PCF290ZI; Olympus Corp., Tokyo, Japan) easily passed through the hernia ostium and was inserted into the sigmoid colon within the hernia sac. A circumferential type 3 tumor was confirmed in the same area and a biopsy was performed (Fig. 2). However, we were unable to insert the colorectal tube as neither the scope nor the guidewire could be advanced past the tumor into the proximal colon.

The pathological diagnosis identified the tumor as adenocarcinoma (tub1 > tub2). Contrast-enhanced CT revealed no invasion into the surrounding tissues (hernia sac, spermatic cord, or testis) and identified two enlarged paracolic lymph nodes (12 mm) and intermediate lymph nodes (10 mm). There were no distant metastases to the lungs or liver. Based on these findings, the patient was diagnosed with sigmoid colon cancer within a left inguinal hernia, staged as cT4aN1bM0, stage IIIb according to the 8th edition of the Union for International Cancer Control (UICC) TNM classification.

The treatment approach was explained to the patient as follows: advanced colon cancer is typically treated through tumor removal, including lymph node dissection, followed by suturing the intestinal tracts together in a single surgery. However, for patients with Incomplete bowel obstruction like you, a temporary ileostomy is often created to reduce the risk of suture failure. This ileostomy is then closed in a second surgery on another date. We proposed this surgical procedure. Given the patient's strong preference for a single surgery, we re-proposed Hartmann procedure and non-mesh hernia repair simultaneously. The planned surgical procedure included the following steps: first, gaining access to the abdominal cavity via a lower median incision; second, releasing the incarcerated sigmoid colon from the hernia sac; third, performing an oncological sigmoid colectomy including lymph node dissection and colostomy; and finally, repairing the hernia using a non-mesh approach through an inguinal incision.

**Fig.3:**
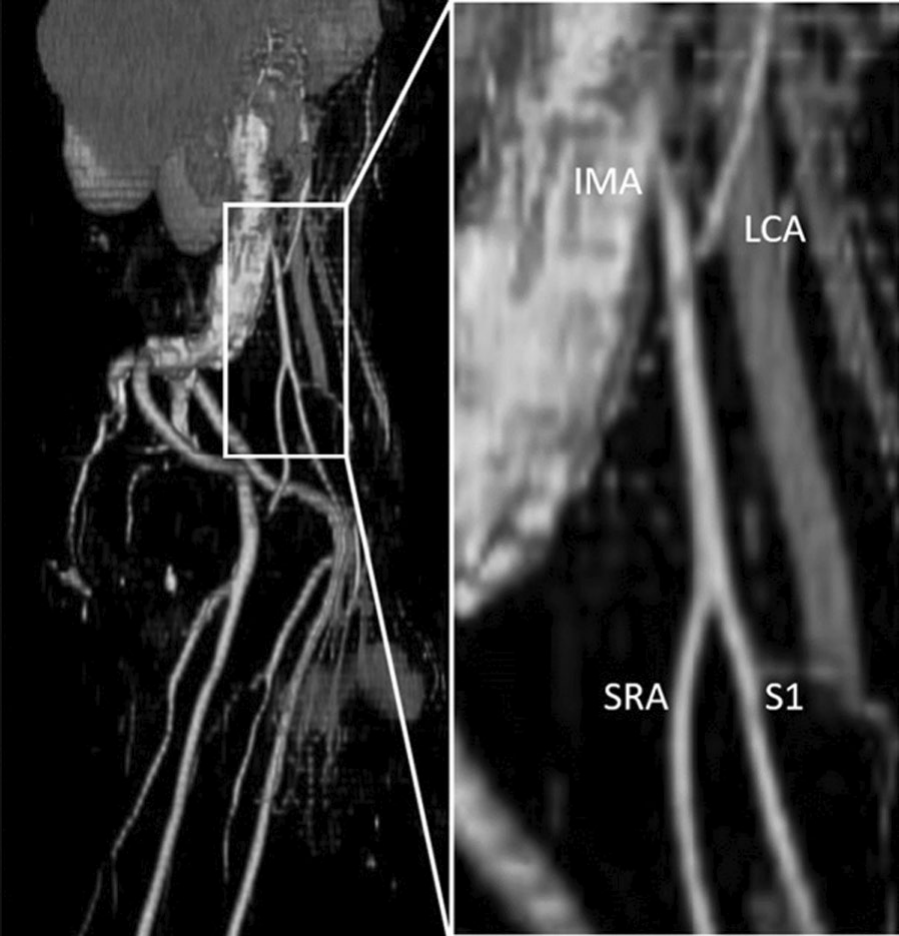
Reconstructed arteria image based on contrast-enhanced CT. Sigmoid artery (S1) proceeded the hernia sac after branching from IMA to the LCA and then to the SRA

**Fig.4:**
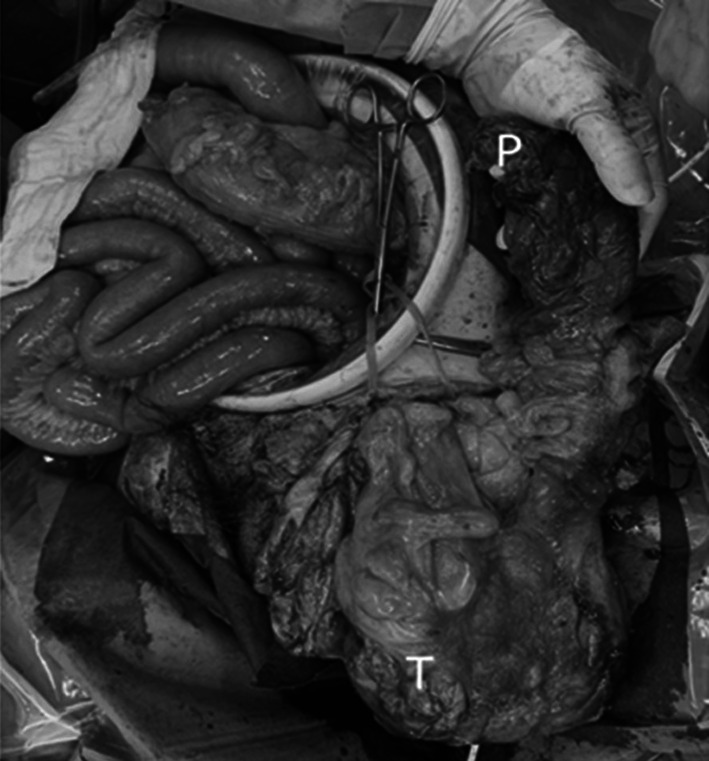
Tumor (T) and proximal stump of sigmoid colon (P) have prolapsed out of the abdominal cavity from the inguinal incision

We performed the surgery on the tenth day after admission. An incision was made in the lower abdomen, and the sigmoid colon was pulled from the abdominal cavity, which was inserted into the left inguinal hernia; however, we were unable to release the intestinal tract. Therefore, to remove the fecal mass in the intestinal tract within the hernial sac, we transected the intestinal tract approximately 10 cm proximal to the hernia and inserted a 5-cm-diameter tube. Subsequently, we successfully evacuated the intestinal contents through the tube; however, we were unable to remove the intestinal tract from the left inguinal hernia. Therefore, we decided to remove the tumor via the inguinal side, and then mesentery of sigmoid colon including blood vessels and lymph nodes from the abdominal side. However, since the mesentery of sigmoid colon was bundled with the hernia orifice as its apex, the blood vessels within the mesentery, including the inferior mesenteric artery (IMA), showed deviation. We confirmed the course of the IMA, sigmoid artery, and superior rectal artery (SRA) by referring to the reconstructed images from contrast-enhanced CT performed before surgery (Fig. 3). Considering the location of the enlarged lymph nodes (para-intestinal and intermediate lymph nodes), we decided on the resection line to cut only the S1 branching from the SRA and preserve the SRA. Dorsal sigmoid resection was not performed. A second incision was made on the inguinal side. After opening the inguinal canal and hernia sac, we confirmed the presence of the sigmoid colon within the hernia sac. We performed dissection around the tumor and were able to preserve the vas deferens and testicles. After the proximal intestinal tract was retracted to the inguinal side, the sigmoid mesocolon was resected (Fig. 4). The intestinal tract was excised from the abdominal side, and the specimen was removed from the inguinal side. Single-hole colostomy was performed on the left lower abdomen. The hernia was repaired using the Iliopubic Tract Repair method without a mesh. The operation time was 5 h and 47 min, and the blood loss was 647 mL.

The pathological results are as follows. No cancer invasion was observed into the peritoneum of the hernia sac (T4a), and no lymph node metastasis was observed. We diagnosed sigmoid colon cancer type 1, 75 × 45 mm, pT4a N0M0 stage IIb, according to the UICC 8th ed. TNM classification. The patient developed pancreatitis during the postoperative course. However, he showed improvement with conservative treatment and was discharged on the 17th hospital day. At 8 months after the surgery, the patient has shown no recurrence of the hernia or cancer.

## Discussion

Cases of primary colonic malignancy (colon cancer) in the inguinal hernia sac are rare. Since 2000, 24 cases, including the present case, have been reported in the English literature (Table 1). All patients were old aged men. We speculate that this is because inguinal hernia and colon cancer are both more common in older men. Fourteen reports described the duration of the hernia problems, of which eight cases (more than half) involved hernia problems lasting for more than 10 years. Although the course of the hernia was reducible at the time of onset, at some point, it became irreducible and the patient came to the hospital or the hernia became incarcerated after becoming irreducible. Tan et al. [1] suggested that the tumor mass may play a role in hernia incarceration. If the sigmoid and transverse colon, which are not anchored to the retroperitoneum, are involved, they invaginate more easily into the hernia sac. Among the 24 reported cases, 19 cases (79%) had sigmoid or transverse colon incarceration. Among these, 17 cases (71%) showed incarceration of the sigmoid colon in the left inguinal hernia, which are anatomically close to each other. Although previous studies have not clarified whether hernia or cancer develops first, the prevailing opinion is that the two have no causal relationship [2].

**Table 1:** Reported cases of primary colonic malignancy (colon cancer) in the inguinal hernia sac since 2000

Group	References			Patient characteristics				Preoperative diagnosis					
	Year	Author	Number	Age	Sex	Hernia side	Hernia onset (years ago)	Tumor location	Duration to surgery	Perforation/infection	Bowel obstruction	Anemia	Colonoscopy
Ｐ	2003	Kouraklis G	8	79	M	L	N/A	S	0	＋	＋	N/A	−
	2010	Ko KH	7	84	M	L	20	S	0	＋	＋	＋	−
	2013	Tan A	6	65	M	L	Long reducible	S	0	＋	−	N/A	−
	2016	Kulasegaran S	10	66	M	L	N/A	S	0	＋	−	N/A	−
	2016	Diao K	9	48	M	L	N/A	T	0	＋	−	＋	−
	2019	Mizuno H	5	73	M	L	N/A	S	0	＋	−	＋	−
	2020	Sabra H	4	87	M	L	N/A	S	0	＋	−	N/A	−
	2023	Chida K	3	80	M	L	N/A	S	0	＋	−	＋	−
B	2003	Cervinka A	12	69	M	L	15	N/A	N/A	−	＋	N/A	−
	2003	Tan GY	11	62	M	L	6	S	0	−	＋	＋	−
	2007	Tan SP	1	69	M	L	N/A	T	N/A	−	＋	N/A	−
	2023	president		79	M	L	20	S	10	−	＋	＋	＋
O	2009	Ruiz-Tovar J	19	67	M	L	N/A	S	N/A	−	−	＋	＋
	2011	Pernazza G	21	70	M	R	20	C	N/A	−	−	＋	＋
	2012	Carr WR	18	65	M	L	Some months	S	N/A	−	−	＋	＋
	2014	Marsden M	24	78	M	R	3	C	N/A	−	−	＋	−
	2014	Kanemura T	20	67	M	L	3	S	N/A	−	−	＋	＋
	2016	Daly D	22	81	M	L	5	S	N/A	−	−	＋	−
	2017	Sharma SK	17	84	M	R	N/A	A	N/A	−	−	＋	−
	2018	Chern TY	16	83	M	R	10	A	N/A	−	−	＋	＋
	2020	Baldi D	15	88	M	L	N/A	S	N/A	−	−	＋	＋
	2021	Gerosa M	14	79	M	L	N/A	S	N/A	−	−	N/A	＋
	2021	Grossi U	23	61	M	L	10	S	N/A	−	−	N/A	＋
	2022	Zhang J	13	70	M	R	20	S	40	−	−	N/A	＋

Group	Operative procedure for hernia								
	**Open/laparoscopy**	Incision	Surgical approach	Release of incarcerated colon	Operation	Varicocele orchiectomy	Initial surgery	Mesh	2nd surgery
P	Open	Inguinoscrotal	Groin	−	Sigmoidectomy＋double orifices colostomy	−	Not complied with	−	Interrupted suture with mesh
	Open	Inguinoscrotal	Combine	−	Sigmoidectomy (Hartmann procedure)	−	Primary closure	−	
	Open	Midline laparotomy	Combine	−	Sigmoidectomy (Hartmann procedure)	−	Primary closure	−	
	Open	Inguinoscrotal	Combine	−	Sigmoidectomy (Hartmann procedure)	−	Primary closure	−	
	Open	Inguinoscrotal	Groin	−	Transverse colectomy	−	Primary closure	−	
	Open	Inguinoscrotal	Combine	−	Sigmoidectomy	−	Marcy	−	
	Open	Inguinoscrotal	Groin	−	Sigmoidectomy (Hartmann procedure)	−	Primary closure	−	
	Open	Inguinoscrotal	Combine	−	Sigmoidectomy (Hartmann procedure)	−	Primary closure	−	Direct Kugel (mesh)
B	Open	Inguinoscrotal	Combine	−	Colectomy ＋T colostomy	−	Bassini	−	
	Open	Inguinoscrotal	Combine	−	Sigmoidectomy	−	Posterior mesh repair	＋	
	Open	Midline laparotomy	Combine	＋	Right hemicolectomy	−	N/A	N/A	
	Open	Midline laparotomy	Combine	−	Sigmoidectomy (Hartmann procedure)	−	Iliopubic tract repair	−	
O	Open	Inguinoscrotal	Combine	＋	Sigmoidectomy	−	Lichtenstein	＋	
	Laparoscopic	Navel	Lap only	Pneumoperitoneum	Right hemicolectomy	−	Primary closure	−	
	Laparoscopic	Navel	Lap only	Pneumoperitoneum	Sigmoidectomy	−	Lichtenstein	＋	
	Laparoscopic→Open	Navel	Combine	−	Right hemicolectomy	−	Shouldice repair	−	
	Laparoscopic	Navel	Lap only	Release adhesion	Sigmoidectomy	−	High ligation	−	
	Open	Midline laparotomy	Combine	−	Sigmoidectomy	＋	Posterior mesh repair	＋	
	Open	Midline laparotomy	Abdominal	＋	Right hemicolectomy	＋	Primary closure	−	
	Laparoscopic	Navel	Lap only	Release adhesion	Right hemicolectomy	−	Laparoscopic defect closure	−	
	Open	Midline laparotomy	Combine	Open sac	Sigmoidectomy	＋	N/A	N/A	
	Laparoscopic	Navel	Lap only	Pneumoperitoneum	Sigmoidectomy (Hartmann procedure)	−	Not complied with	−	
	Laparoscopic	Navel	Combine	−	Sigmoidectomy	−	N/A	N/A	
	Laparoscopic→Open	Navel	Combine	−	Sigmoidectomy	−	Bassini	−	

CT is the most useful imaging examination for diagnosing the presence of cancer within the hernial sac and provides information on local progression, lymph node metastasis, and distant metastasis. Furthermore, it can indicate the presence of perforation or abscess formation and the degree of intestinal obstruction. These findings are useful for understanding medical conditions that are directly linked to treatment. Based on diagnosis at visit, we divided 24 cases into the perforation and abscess formation group (group P), the bowel obstruction without infection group (group B), and the group showing only non-reduction of the herniated intestine (group O). All eight patients in group P underwent emergency surgery [3–10]. Among the four patients in group B (including the patient in the present case) [1, 11, 12] and 12 patients in group O [13–24], 10 underwent preoperative colonoscopy, which was not performed in group P. Regarding the usefulness of colonoscopy, Baldi et al. [15] reported that malignancy could be diagnosed before surgery. Gerosa et al. [14] stated that by diagnosing rare neuroendocrine cancers preoperatively, they were able to provide appropriate treatment for a condition with poor prognosis. In addition, Marsden et al. [24] performed colonoscopy in a bilateral hernia patient showing intestinal prolapse and diagnosed cecal cancer within the right hernial sac. On the other hand, Abe et al. [25] advanced the scope beyond the hernia sac to the descending colon in a case in which sigmoid colon cancer was found on the dorsal side of the hernia sac and reported that the endoscope was incarcerated in the hernial ostium upon removal. We attempted to insert a colorectal tube into the intestinal tract proximal to the cancer site, but this was not possible. If it had been inserted, it would have crossed the hernia gate twice. As previously reported [25], the risk of incarceration at the hernia ostium during removal should be taken into consideration.

Regarding hernia incarceration due to colon cancer, the procedure differs depending on individual medical condition. When it is an elective surgery like in the present case, laparoscopic approach is standard. The reasons why we chose open surgery are follows. To complete the sigmoid colectomy laparoscopically, release of incarceration is necessary. Therefore, we attempted to remove the bowel obstruction using a transanal drainage. But it was unsuccessful. After that, we tried to alleviate the bowel obstruction by fasting and taking laxatives, but there was almost no change. As a result, laparotomy was chosen. After laparotomy, we attempted to remove the incarceration to facilitate oncological cancer resection. First, the colon proximal to the tumor was incised, and direct drained. However, the incarceration was not removed, so we added inguinal incision. We also evaluated the surgical techniques by group. In group P, in all cases except one [6], an incision was made in the groin to treat the contaminated sac. If the contamination is limited to the hernia sac, the prognosis is generally good [3, 4, 6, 8, 9]. However, Fournier's gangrene may develop from an infection of the scrotum, even if the contamination is localized within the hernia sac [7], or the cancer may invade the hernia sac (peritoneum) and perforate the extraperitoneal cavity (subcutaneous tissue) [5]. The rate of intestinal anastomosis varied greatly depending on the condition of the patient at the time of presentation: 25% (2/8 patients) in group P, 75% (3/4 patients) in group B, and 92% (11/12 patients) in group O. In the present case, since intestinal anastomosis was feasible, we initially proposed a two-stage surgery. However, we ultimately opted for the Hartmann procedure because the patient did not consent to a temporary ileostomy. Gerosa et al. [14] reported the only case that involved the Hartmann procedure in the group O. Since their case involved a large-cell neuroendocrine carcinoma, which has an extremely poor prognosis, this procedure may have been performed due to the need to start postoperative adjuvant chemotherapy immediately after the surgery. Thus, the choice of the surgical method is influenced by factors other than the pathology of the patient at the visit. Oncologic resection of the colonic mesentery (lymph node dissection) is commonly agreed to be fundamental to the extent of cancer resection. For this purpose, it is desirable to remove the incarceration first. In group P, the mesentery was removed from the inguinal side without releasing the hernia incarceration for infection control (release rate: 0%). In group B, only Tan et al. [1] succeeded in releasing the hernia incarceration after incision of the external oblique muscle from the inguinal side (release rate: 25%). In group O, release of hernia incarceration was possible in eight of the 12 patients (67%), showing a similar trend to the rate of bowel anastomosis. A notable factor is the timing of the release during laparoscopic surgery. In three cases, release occurred immediately after pneumoperitoneum, while in two patients, it was achieved by simple adhesion detachment after pneumoperitoneum. We assumed that air entering the hernial sac pushed the intestinal tract out of the side of the sac. Mesenteric resection is usually performed from the inguinal side if the hernial incarceration cannot be released. Daly et al. [22] performed D2 dissection from the abdominal side using open surgery, and Grossi et al. [23] performed ligation of the inferior mesenteric artery and vein and mobilization of the intestinal tract using laparoscopic surgery. In our case, the course of the blood vessels in the mesentery could not be easily confirmed before the release. We used contrast-enhanced CT-reconstructed images to determine the general location of the blood vessels and lymph nodes and thereby determine the mesenteric transection line. This disease represents a special condition in which cancer resides within the hernia sac and involves the spermatic cord and testis. Of the 24 previously reported cases, varicocele orchiectomy was performed in three cases [15, 17, 22]. Since these three cases were in group O, we believe that resection was performed to achieve a more curative outcome.

Another point that requires discussion is the hernia repair procedure. In group P, the patients essentially underwent primary closure, and two of them [1, 17] underwent secondary radical surgery with a mesh. Even in the cases with infection in groups B and O, a mesh was used in only 4 of 13 cases (31%) (this technique has only been described in the literature). This may be because surgery involving intestinal resection was classified as Class II (group P, group O) or III (group P) by the National Nosocomial Infections Surveillance System [26]. The inguinal region is also a possible site of cancer recurrence. Contrast-enhanced CT or positron emission tomography CT is usually performed to differentiate between hernia and cancer recurrence. Regarding fluorodeoxyglucose uptake by the surgical mesh, Bahçeci et al. [27] reported that a 5-year-old mesh showed positive results, raising concerns related to false-positive findings in patients with cancer. Kudou et al. [28] reported that when laparoscopy was performed to review a case of peritoneal dissemination of colorectal cancer, cancer seeding nodules were found only at the site of bilateral inguinal hernia repair (mesh plug technique) performed 6 years earlier (no dissemination to other sites or ascites was observed). Murphy et al. [29] reported that tumor adhesion is enhanced at sites of persistent inflammation. Thus, the use of meshes in the inguinal region, which is prone to infection and is a potential site of recurrence, is associated with notable limitations.

## Conclusions

We encountered a case of Incomplete bowel obstruction caused by sigmoid colon cancer arising from an inguinal hernia. In previous reports, the pathophysiology at the onset varied from perforation and strangulation to intestinal obstruction and other complications. Although the surgical technique depends on the patient's condition, the Hartmann procedure is frequently performed in cases involving infection, and intestinal anastomosis is frequently performed through an inguinal incision in cases involving intestinal obstruction. Laparoscopic surgery was performed in the absence of infection or obstruction. In contrast, most hernias are repaired by initial surgery using a non-mesh method. Although colon cancer within the hernia sac is infrequent, patients who have had inguinal hernias for a long time and exhibit difficulty in hernia release should be examined for potential malignant complications.

## Data Availability

All data related to the outcomes have been included in the manuscript.

## Abbreviations

CT: Computed tomography
UICC: Union for International Cancer Control
IMA: Inferior mesenteric artery
SRA: Superior rectal artery
